# A Pandemic Influenza H1N1 Live Vaccine Based on Modified Vaccinia Ankara Is Highly Immunogenic and Protects Mice in Active and Passive Immunizations

**DOI:** 10.1371/journal.pone.0012217

**Published:** 2010-08-16

**Authors:** Annett Hessel, Michael Schwendinger, Daniela Fritz, Sogue Coulibaly, Georg W. Holzer, Nicolas Sabarth, Otfried Kistner, Walter Wodal, Astrid Kerschbaum, Helga Savidis-Dacho, Brian A. Crowe, Thomas R. Kreil, P. Noel Barrett, Falko G. Falkner

**Affiliations:** R&D Vaccines, Biomedical Research Center, Baxter BioScience, Orth/Donau, Austria; Louisiana State University, United States of America

## Abstract

**Background:**

The development of novel influenza vaccines inducing a broad immune response is an important objective. The aim of this study was to evaluate live vaccines which induce both strong humoral and cell-mediated immune responses against the novel human pandemic H1N1 influenza virus, and to show protection in a lethal animal challenge model.

**Methodology/Principal Findings:**

For this purpose, the hemagglutinin (HA) and neuraminidase (NA) genes of the influenza A/California/07/2009 (H1N1) strain (CA/07) were inserted into the replication-deficient modified vaccinia Ankara (MVA) virus - a safe poxviral live vector – resulting in MVA-H1-Ca and MVA-N1-Ca vectors. These live vaccines, together with an inactivated whole virus vaccine, were assessed in a lung infection model using immune competent Balb/c mice, and in a lethal challenge model using severe combined immunodeficient (SCID) mice after passive serum transfer from immunized mice. Balb/c mice vaccinated with the MVA-H1-Ca virus or the inactivated vaccine were fully protected from lung infection after challenge with the influenza H1N1 wild-type strain, while the neuraminidase virus MVA-N1-Ca induced only partial protection. The live vaccines were already protective after a single dose and induced substantial amounts of neutralizing antibodies and of interferon-γ-secreting (IFN-γ) CD4- and CD8 T-cells in lungs and spleens. In the lungs, a rapid increase of HA-specific CD4- and CD8 T cells was observed in vaccinated mice shortly after challenge with influenza swine flu virus, which probably contributes to the strong inhibition of pulmonary viral replication observed. In addition, passive transfer of antisera raised in MVA-H1-Ca vaccinated immune-competent mice protected SCID mice from lethal challenge with the CA/07 wild-type virus.

**Conclusions/Significance:**

The non-replicating MVA-based H1N1 live vaccines induce a broad protective immune response and are promising vaccine candidates for pandemic influenza.

## Introduction

Influenza virus infection is a non-eradicable zoonosis and therefore pandemics caused by novel influenza A subtypes are a permanent threat (for review see: [1]). Despite the emergence and spread of the highly pathogenic avian H5N1 virus since 1997 and the absence of H2 strains from human circulation since 1968, the first pandemic of this century was not caused by H5 or H2 subtypes but by the novel swine-origin H1N1 strains first detected in humans in April 2009. The global spread of the novel H1N1 influenza subtype has made the development of vaccines a global public health priority. Several strategies are currently being followed to produce pandemic vaccines, such as the development of inactivated whole virus vaccines, subunit vaccines, recombinant viral proteins and live vaccines. Vaccines based on inactivated influenza virus are usually derived from embryonated hens' eggs or, more recently, from permanent cell cultures. Protective immunity elicited by these vaccines is mainly based on neutralizing antibodies directed against the HA (reviews: [2], [3]).

However, a more broad immune response which includes efficient antibodies against the influenza surface proteins as well as induction of CD8 T cells – as accomplished by live vaccines - would be desirable. Attenuated live vaccines such as cold-adapted influenza strains [4], [5] or nonreplicating, NS-1 gene-deleted influenza virus [6], [7] presumably have these advantages. Intranasal application of such pre-pandemic live vaccines might, however, result in new reassortant strains by co-infections in the respiratory tract with wild-type influenza strains, thereby raising safety concerns. Moreover, in certain instances, influenza reassortants of the cold-adapted internal gene backbone with avian strains have been shown to have incompatible gene segments and induce only subpotent immune responses [8]. Only the re-introduction of the polybasic cleavage site into the HA (previously deleted to attenuate the live virus) restored infectivity and immunogenicity [9]. In another case, passaging of the live vaccine in host cells was required to achieve acceptable growth. Passaging, however, may result in reduced immunogenicity that may require screening of adequate reassortants [8]. Finally, the long-term effect of repeated intranasal administration of high doses of live virus vaccines on the olfactory system is largely unknown.

To circumvent these issues, live vaccines based on nonreplicating poxviral vectors - such as the highly attenuated MVA vector – are a promising alternative. These vectors have a long-standing safety record, induce excellent T cell responses and are usually administered by reliable subcutaneous or intramuscular routes. The purpose of this study was to evaluate the immune response and the protective potential of MVA-based influenza vaccines expressing the protective antigens hemagglutinin and neuraminidase of the novel H1N1 strain. Efficient induction of antibodies and surprisingly high levels of CD8 T cells were induced against both antigens.

## Materials and Methods

### Ethics statement

All animal experiments were reviewed by the Institutional Animal Care and Use Committee (IACUC) and approved by the Austrian regulatory authorities. All animal experiments were conducted in accordance with Austrian laws on animal experimentation and guidelines set out by the Association for Assessment and Accreditation of Laboratory Animal Care (AAALAC). Animals were housed in facilities accredited by the AAALAC.

### Cells and viruses

The Vero (CCL-81) and DF-1 (CRL-12203) cell lines were obtained from the American Type Culture Collection. They were cultivated in DMEM (Biochrom AG) containing 5% fetal calf serum (FCS). Chicken embryo cells (CEC) were cultivated in M199 (Gibco, Inc.) containing 5% fetal calf serum (FCS). Madin-Darby canine kidney (MDCK) cells were maintained serum free in Ultra-MDCK medium (Bio Whittaker). The influenza virus A/California/07/2009 (H1N1; CDC#2009712112) was kindly provided by the Centers for Disease Control and Prevention (CDC, Atlanta, USA). The vaccinia virus strain Lister/Elstree (VR-862) was obtained from the American Type Culture Collection. The basis of the Lister constructs was the subcloned virus vpDW-862/Elstree [10]. The MVA strain (MVA 1974/NIH clone 1) was kindly provided by B. Moss (National Institutes of Health).

### Cloning and sequencing of hemagglutinin and neuraminidase genes

The hemagglutinin (Genbank #FJ966082) and neuraminidase (Genbank #FJ966084) sequences of A/California/7/2009 were synthesized (Geneart, Regensburg, Germany). Both synthetic genes are driven by the strong early/late vaccinia virus promoter mH5 and terminate with a vaccinia virus specific stop signal downstream of the coding region that is absent internally. Both expression cassettes were cloned into the MVA transfer plasmid pHA-vA [11] resulting in pHA-mH5-H1-Ca and pHA-mH5-N1-Ca, respectively. The insertion plasmids direct the gene cassettes into the MVA HA-locus, close to deletion III of MVA [12], [13]. Both gene cassettes were inserted in parallel into the vaccinia transfer plasmid pER-mH5-PL resulting in pER-mH5-H1-Ca or pER-mH5-N1-Ca, respectively. The plasmid pER-mH5-PL was obtained by insertion of the vaccinia virus promoter mH5, a vaccinia virus stop signal (TTTTTNT) and a multiple cloning site (StuI, NcoI, PvuII, SpeI, HindIII, SacI, XmaI, SalI, NotI) into plasmid pER that directs the gene cassette into the D4/D5 intergenic region [14].

### Construction of recombinant vaccinia viruses

#### rVVL-H1-Ca and rVVL-N1-Ca

Twenty micrograms of pER-mH5-H1-Ca or pER-mH5-N1-Ca plasmid DNA were transfected into vaccinia virus Lister-infected Vero cells by calcium phosphate co-precipitation and further processed as described previously [14]. Plaque isolates were purified three times and expanded for large scale preparations in Vero cells. The vaccinia virus stocks were prepared in Vero cells by infection with 0.1 MOI for 72 hours. Infected cells were harvested and sucrose cushion purified viral stocks were prepared [14].

#### MVA-H1-Ca and MVA-N1-Ca

Twenty micrograms of pHA-mH5-H1-Ca or pHA-mH5-N1-Ca plasmid DNA were transfected into MVA infected CEC by calcium phosphate precipitation and further processed as described previously [11]. The purified recombinant virus isolates were expanded for large scale preparations in CEC.

### Western blot analysis

Expression of the H1 or N1 proteins by recombinant vaccinia viruses was detected by Western blotting. Vero cells in case of the VV-L constructs, or the avian cell line DF-1 [15] in case of MVA, were infected at a multiplicity of infection of 0.1 for 48 h. MVA-H1-Ca or rVVL-H1-Ca infected cells were harvested by scraping or by adding trypsin. MVA-N1-Ca or rVVL-N1-Ca infected cells were harvested by scraping. Sonicated cell lysates were loaded onto 12% polyacrylamide gels (BioRad, Inc) and afterwards blotted on nitrocellulose membrane (Invitrogen, Inc). To detect the H1 protein, a sheep antiserum against the A/California/7/2009 hemagglutinin (NIBSC 09/152) was used. Donkey-anti-sheep alkaline phosphatase-conjugated IgG (Sigma Inc.) was used as a secondary antibody. To detect the N1 protein a polyclonal rabbit anti avian influenza A neuramindase (Abcam, ab21304) was utilized. Goat-anti-rabbit alkaline phosphatase-conjugated IgG (Sigma Inc.) was used as a secondary antibody. A whole virus vaccine H1N1 A/California/7/2009 [16] served as positive control.

### Immunizations of Balb/c mice for analysis of humoral immunity

Groups of 6 female Balb/c mice (8–10 weeks old) were immunized intramuscularly either once (day 0) or twice (days 0 and 21) with 10^6^ or 10^7^ pfu of recombinant MVA-H1-Ca or rVV-L-H1-Ca. Control groups were immunized with 10^7^ pfu wild-type MVA, 10^7^ pfu wild type VV-L, 3.75 µg of whole virus vaccine H1N1 A/California/7/2009 (inact. H1N1) or with PBS buffer. Mice were challenged intranasally with 10^5^ TCID50 per animal of A/California/7/2009 on day 42 and lungs were removed three days later and frozen at ≤60°C for virus titer determination. Blood samples were taken to measure HA-specific IgG concentration and HI titer on days 20 and 41.

### Immunizations of Balb/c mice for analysis of cellular immunity

Groups of 6 female Balb/c mice (8–10 weeks old) were immunized intramuscularly on days 0 and 21 with 10^6^ pfu of recombinant MVA-H1-Ca or rVVL-H1-Ca, wild-type MVA or VV-L, 3.75 µg of whole virus vaccine H1N1 A/California/7/2009 (inact. H1N1) or with PBS buffer. Spleens were obtained for IFN-γ analyses at day 28 post-immunization. Furthermore, groups of 5 mice immunized two times with 10^6^ pfu of recombinant MVA-H1-Ca, rVVLH1-Ca, or MVAwt were challenged intranasally with 10^5^ TCID50 per animal of A/California/7/2009 on day 42, and lungs and spleens were collected after euthanizing the mice 7 days after the booster immunization (day 28), at the time of challenge (day 41) or three days thereafter (day 45) for determination of influenza-specific T-cell frequencies.

### Preparation of the lung samples for virus titration

Mice were euthanized and lungs were removed on day 3 post challenge with wild-type H1N1 virus. These tissue samples were stored at <−60°C until they were transferred into homogenization tubes (Precellys Ceramic Kit 2.8 mm, PEQLAB Biotechnologie GmbH) containing 1 ml cell medium supplemented with antibiotics. The lungs were homogenized two times at 5000 rpm for twenty seconds with 5 seconds pause between the intervals with a tissue homogenizer (Precellys24, PEQLAB Biotechnologie GmbH). The infectious H1N1 virus titer in homogenized lung samples was determined by a TCID50 assay performed by titration on Madin-Darby canine kidney (MDCK) cells by serial ten-fold dilutions of samples as described [17].

### Passage assay of low-titer lung samples in MDCK cells

Lung samples with titers ≤3 log10 TCID50 were verified by this passage assay. Confluent MDCK cells (grown in 75 cm^2^ Roux flasks) were infected with 100 µl of mouse lung sample. The adsorption medium was 10 ml Ultra MDCK-Medium (BioWhittaker) and contained 1 µg/ml TPCK trypsin (Sigma-Aldrich®). After one hour virus adsorption 40 ml Ultra MDCK-Medium supplemented with 1 µg/ml TPCK trypsin was added and the infection was further incubated for 6 days. Afterwards the MDCK cells were analysed for infection. The detection limit of the assay, determined by spiking with 100, 10 and 1 TCID50 virus per flask, was approximately 1 TCID50/ml.

### Determining the lethal challenge dose of H1N1 wt in SCID mice

To define the lethal challenge dose 50 (LD50) of wt H1N1 A/California/7/2009 virus in severe combined immunodeficient (SCID) mice, 4–5 week old female SCID mice (strain CB17/Icr-Prkdcscid/IcrCrl; Charles River, Sulzfeld, Germany) were used. Mice were challenged intranasally (10 µl) with 10-fold serial dilutions of the wt A/California/7/2009 (H1N1) strain. The virus dose that kills 50% of the SCID mice (lethal dose 50, LD_50_) was calculated by the software program Graph-Pad Prism 5.

### H1N1 challenge and passive protection of SCID mice

For generation of sera for passive transfer studies, CD1 mice (Charles River) were immunized twice (d0, d21) with 10^6^ pfu recombinant MVA-H1-Ca, rVVL-H1-Ca, MVA wt or 3.75 µg of whole virus vaccines H1N1 A/California/07/2009 (inact. H1N1), respectively. Serum pools were prepared on day 42 and analysed via HI and ELISA. For passive protection experiments, 200 µl of the produced sera were injected intraperitoneally into 4–5 week old SCID mice. One or two days afterwards, mice were challenged by intranasal instillation with 10^5^ TCID50 per animal of the A/California/07/2009(H1N1) wild-type strain and monitored for clinical parameters and survival for 30 days.

### Hemagglutination Inhibition (HI) Assay

The HI titer of the sera was determined using chicken erythrocytes as described [17]. Briefly, sera were treated with receptor destroying enzyme, inactivated at 56°C and two-fold serially diluted. Sera were incubated with formalin-inactivated A/California/07/2009 virus standardized to 8 HA units/50 µl [18] followed by incubation with erythrocytes. The detection limit was a HI titer of ≤10.

### Neuraminidase Inhibition (NI) Assay

Anti-neuraminidase serum antibodies were measured with a modified, miniaturized neuraminidase inhibition assay [19]. Briefly, serially diluted inactivated H1N1 A/California/07/2009 wild-type virus preparation was mixed with an equal volume of fetuin [25 mg/mL] and incubated for 18 h at 37°C. After addition of periodate and incubation for 20 min, arsenite was added and the solution mixed until the brown colour disappeared. Thiobarbituric acid was then added and samples boiled for 15 min. The pink reaction product was extracted by butanol and its absorbance determined at 550 nm using a reaction blank as reference. The half-maximal neuraminidase activity (EC50) was determined using non-linear regression of the absorbance data (GraphPad Prism, GraphPad Software, USA). For the subsequent neuraminidase inhibition studies, the concentration of the virus was adjusted to an equivalent of half-maximal neuraminidase activity. Serially diluted sera were incubated with the appropriately diluted virus preparation for 1 h and the neuraminidase activity determined as described above. The neuraminidase inhibition titer was defined as the reciprocal serum dilution at which neuraminidase activity was 50% inhibited.

### Preparation of single cell suspensions from spleens or lungs of immunized and challenged mice

Single cell suspensions of splenocytes were obtained by grinding 5 spleens per group through a metal mesh into culture media (45% RPMI1640 [Gibco], 45% CLICKs Medium [Sigma], 10% FCS [Gibco], Penicillin-Streptomycin [Gibco], 2 mM L-glutamine [Gibco]). Splenocytes were either used immediately or were frozen in Cryostor CS-10 (VWR). Single cell preparations were also obtained from the lungs of immunized mice before and after challenge with H1N1 swine flu virus. After euthanizing the mice, lungs were flushed by injecting 0.5–1 ml of PBS/Heparin (5 IE/ml) into the right ventricle of the heart. The lungs were then removed and immediately placed into culture media, cut into small pieces and digested for 25 min at RT by adding 5 mM MgCl_2_ [Merck], 150 Units/ml DNAse I [Roche] and 1 mg/ml collagenase XI [Sigma] to the culture media. After digestion, the remaining pieces were ground through a metal mesh into culture media, and the resulting cell suspension was filtered through a 70 µm nylon mesh [BD]. Residual red blood cells were removed using RBC lysis buffer [Sigma] according to the manufacturer instructions, and the cells were used immediately for FACS-based IFN-γ analyses.

### T-cell IFN-γ analysis

Peak response frequencies of vaccine-specific IFN-γ producing T-cells in Balb/c mice were determined by flow cytometric intracellular cytokine staining in spleens 7 days after the second immunization, or in lungs on the day before challenge (day 41) or 3 days after challenge (day 45). Approximately 2×10^6^ cells were dispensed into 96-well round-bottom-plates (Costar) and stimulated for approximately 14 h at 37°C with vaccine antigens (H1N1/California/07/2009, H1N1/Brisbane/59/2007, H1N1/New Caledonia/20/99, H5N1/Vietnam/1203/2004) at 3 µg/mL hemagglutinin, or with peptide pools of 15-mers overlapping by 10 amino acids at 2 µg/ml per peptide. Two pools of 111 and 92 peptides were used, spanning the entire H1 or N1 proteins of A/California/07/2009, respectively. Cells incubated with medium alone served as a negative control. After 2 h 10 µg/ml brefeldin A (Sigma) was added to inhibit secretion of cytokines and further incubated for 12 h. Then, cells were resuspended in 50 mM PBS/EDTA, and stained with LIVE/Dead Violet Kit (VIVID, Molecular Probes) diluted 1∶10000 in PBS for 15 min at room temperature. Cells were washed and incubated with rat anti-mouse CD4-APC antibody (BD Biosciences, 0.4 µg/mL), and CD8-APC H7 antibody (BD Biosciences, 1.5 µg/ml) for 10 min at room temperature. After washing and fixation with 1% paraformaldehyde (Merck), cells were permeabilized in PBS supplemented with 0.08% saponin (Sigma), and incubated with rat anti-mouse IFN-γ FITC antibody, (BD Biosciences, 0.5 µg/mL) and rat anti-mouse CD3-PerCP (BD Biosciences, 1.5 µg/mL) for 30 min at room temperature. Finally, cells were washed and fixed with 1% paraformaldehyde. At least 100.000 viable cells were applied on a FACSCanto-2 (BD Biosciences), and data analysis was performed using the FlowJo software (Tree Star, Inc.). Percentages of IFN-γ producing T-cells were calculated after gating on VIVID negative, CD3 positive, CD4 or CD8 positive lymphocytes.

### Microneutralization assay

The microneutralization assay was done as described previously [16]. Briefly, sera was diluted and mixed with the A/California/7/09 virus strain at a concentration of 4.5 log TCID_50_/ml. The mixture was incubated for six days on MDCK monolayer before cells were inspected for cytopathic effects. The neutralizing antibody titer was defined as described [16].

### Statistical analysis

For statistical comparison of lung titers the Tukey test was used. The data of T cell responses were statistically analyzed using the two-way ANOVA. Survival differences between animal groups were analyzed using the Kaplan Meyer log rank test of GraphPad Prism software (San Diego, CA). All differences were considered significant at P values <0.05.

## Results

### 1. Construction and characterization of the MVA viruses

The HA and NA genes of the influenza strain A/California/07/2009 (H1N1) were placed downstream of a strong vaccinia early/late promoter [20] and the resulting plasmids were used to construct the viruses MVA-H1-Ca and MVA-N1-Ca by *in-vivo* recombination techniques using transient marker genes. Replicating control viruses based on the vaccinia Lister strain, rVVL-H1-Ca and rVVL-N1-Ca, respectively, were also constructed (see **Table 1** and Methods). The H1 and N1 genes were synthetic genes optimized for expression in vaccinia virus, lacking internal transcription stop signals [21]. The virus constructs were characterized by PCR for absence of wild-type virus and for presence of the HA and NA gene inserts (data not shown).

**Table 1:** Viruses, vaccine constructs and controls used in the study.

vaccine	inserted flu gene	plasmid	Titer(1) (pfu/ml)
MVA-H1-Ca	hemagglutinin	pHA-mH5-H1-Ca(2)	4.0×10^9^
MVA-N1-Ca	neuraminidase	pHA-mH5-NA-Ca	1.6×10^9^
rVVL-H1-Ca	hemagglutinin	pER-mH5-H1-Ca	4.3×10^9^
rVVL-N1-Ca	neuraminidase	pER-mH5-NA-Ca	7.4×10^9^
VVL-wt(3)	empty vector	n.a.	1.7×10^11^
MVA-wt(4)	empty vector	n.a.	5.0×10^9^
Inact. H1N1(5)	n. a.	n.a.	n. a.

(1) titers of sucrose-purified virus preparations (see Methods);

(2) influenza genes in VV constructs are controlled by the vaccinia mH5 promoter; HA and NA genes are derived from the influenza CA/07 strain;

(3) wild-type vaccinia Lister strain;

(4) wild-type MVA (NIH74 LVD clone 6);

(5) inactivated whole virus vaccine based on CA/07 strain; n. a., not applicable.

**Figure 1:**
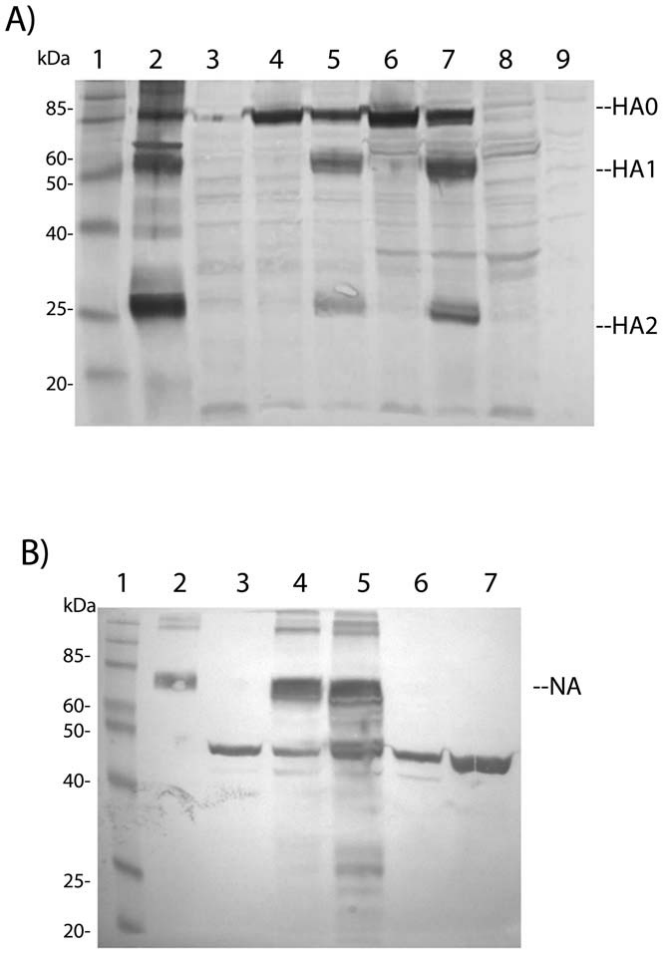
Western blots of chicken cell (DF-1) lysates probed for influenza antigens. (A) Hemagglutinin expression in chicken cells. Lane 1, marker size in kDa. Lane 2, formalin-inactivated purified H1N1 influenza virus. Lane 3, negative control, DF-1 cells infected with wt vaccinia virus. Lane 4, replicating virus rVVL-H1-Ca, no trypsin treatment. Lane 5, replicating virus rVVL-H1-CA, with trypsin treatment. Lane 6, MVA-H1-Ca, no trypsin treatment. Lane 7, MVA-H1-Ca, with trypsin treatment. Lane 8, negative control lysate, MVA wt virus. Lane 9, uninfected cell lysate. HA0, unprocessed hemagglutinin; HA1 and HA2, the two processed HA subunits. B) Neuraminidase expression in chicken cells. Lanes 1-3, samples as above. Lane 4, replicating virus rVVL-N1-Ca. Lane 5, MVA-N1-Ca. Lane 6 and 7, negative controls, lysates of MVA wt virus and non-infected DF-1 cells. The band around 75 kDa represents the neuraminidase (NA).

Next, expression of the influenza genes in avian cells was analyzed. Since parameters such as temporal order and level of gene expression and processing of the protective antigen influence immune responses in vaccinia-based live vaccines, western blot analyses with lysates of infected cells were performed. In infected target cells of the respiratory tract, the influenza hemagglutinin precursor (HA0) is cleaved by intracellular proteases into the heterodimeric receptor molecule (HA1 and HA2) resulting in full infectivity of influenza virus. In order to assess HA antigen levels and processing, expression experiments in MVA-permissive avian cells were performed. Total cell lysates were analyzed by PAGE and Western blotting using anti-hemagglutinin sera. As shown in **Fig. 1A**, the viruses with HA gene inserts induced high level expression of HA in avian DF-1 cells. The large band at around 80 kDa represents the HA0 hemagglutinin-precursor, which is not cleaved in cells lacking the required proteases such as the chicken cells used to propagate MVA (lanes 4 and 6). To further characterize the HA0 precursor, the cell lysates were treated with trypsin (see Methods). In both, the rVVL-H1-Ca and the MVA-H1-Ca infected cells, two novel bands at approximately 64 and 26 kDa appeared that represent the HA1 and HA2 subunits (lanes 5 and 7). The polypeptide pattern of the inactivated whole virus vaccine is shown in lane 2. The HA-specific bands in this control co-migrate with the ones expressed by the viral vectors. In the negative controls these bands are absent (lanes 3, 8, 9). In mouse muscle cells, infected with the same HA containing vectors and controls, the HA0 band could be seen, that was cleaved into the two subunits only upon trypsin treatment. Thus, the same expression pattern was obtained in MVA-permissive avian cells and in non-permissive murine cells that presumably reflect the situation in mice vaccinated by the intramuscular route (data not shown).

To reveal the expression pattern of the neuraminidase constructs, lysates of infected DF-1 cells together with controls, were subjected to Western blotting and probed with an antibody raised against a peptide present in the neuraminidases of different subtypes including N1 and N5 (see Methods). The recombinant vectors induced a broad novel band in the 75 kDa range, representing the highly glycosylated neuraminidase of the A/California/07/2009 (CA/07) strain (**Fig. 1B**, lanes 4 and 5), that was also detectable in the inactivated vaccine preparation (lane 2), but was absent in the negative controls including non-infected and wild-type virus infected cells (lanes 3, 6, 7). The prominent band in the 47 kDa size range (lanes 3–7) is a protein present in DF-1 cells non-specifically cross-reacting with the first antibody.

### 2. Protection studies in immune competent mice

**Figure 2:**
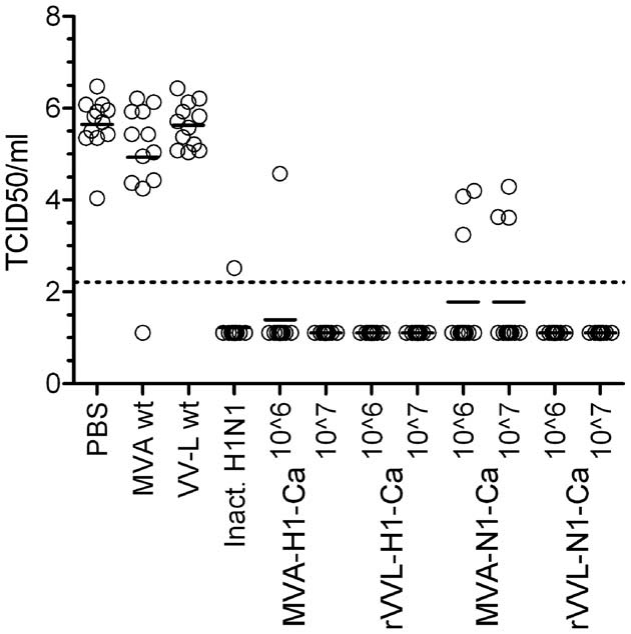
Lung titers in Balb/c mice. The dots represent the lung titers of individual mice vaccinated with the different experimental vaccines or control preparations. Mice vaccinated with the controls (PBS, wild-type MVA (MVA wt) or Lister (VV-L wt) were not protected showing average log10 TCID50 titers of 5.2, 4.9 and 5.4, respectively. Mice vaccinated with inactivated vaccine (inact. H1N1), or two different doses (10^6^, 10^7^ pfu) of MVA-H1-Ca or rVVL-H1-Ca were close to or fully protected. Partial protection was achieved with two dosages of the MVA neuraminidase viruses (MVA-N1-Ca), while full protection was seen with the Lister-based neuraminidase virus (rVVL-N1-Ca). The dotted line represents the detection limit of log_10_ 2.21. Low titers in range of log10<2.21 to 3.0 were confirmed by a passage assay of the lung samples in MDCK cells.

Protection studies were subsequently carried out in BALB/c mice. Upon challenge with H1N1 swine flu virus, this mouse strain develops signs of disease, nevertheless the mice recover after two weeks [22]. Protection from virus replication in the lungs in this model was used as the read-out for efficacy. In the protection experiments performed, the hemagglutinin- and neuraminidase expressing viral constructs were compared to a formalin-inactivated whole virus vaccine (inact. H1N1) [16]. The live vaccines were given by intramuscular injection of 1×10^6^ and 1×10^7^ pfu per animal. The dose of the inactivated vaccine was 3.75 µg (**Table 2**). The groups were boosted after 21 days and all groups were challenged at day 42. Groups of mice immunized with the empty vectors (MVA-wt and VVL-wt) and with phosphate buffered saline (PBS) served as negative controls. The challenge virus was given intranasally at a dose of 1×10^5^ TCID50 per animal. Three days after challenge the lungs were removed and virus titers were determined. The results of the lung titrations are shown in **Fig. 2** and in **Table 2**. In the groups immunized with the hemagglutinin constructs, MVA-H1-Ca or rVVL-H1-Ca, protection from lung virus was almost complete; 92–100% protection was achieved, depending on dose and construct (Table 2, groups 1–4). This protection was significantly higher compared to the control groups immunized with PBS or empty vectors (P<0.0001). Hemagglutinin-inhibition (HI) titers of the mouse sera, performed using the purified swine flu virus as hemagglutinin, were in the range of 453–905. Neutralization titers were also high, i.e. in the range of 761–1076.

**Table 2:** Protection of mice from lung viremia and serology results after **two** vaccinations.

Gr.	Virus	Vaccine dose [pfu or µg/mouse]	Protection n/n total (%)	HI-Titer(1) [GMT(2)]	µNT(3) [GMT(2)]	NI-Titer(4) [GMT(2)]
1	MVA-H1-Ca	10^6^	11/12(5) (92)	453	905	n.d.
2	MVA-H1-Ca	10^7^	12/12 (100)	905	1076	n.d.
3	rVVL-H1-Ca	10^6^	12/12 (100)	453	761	n.d.
4	rVVL-H1-Ca	10^7^	12/12 (100)	640	1076	n.d.
5	MVA-N1-Ca	10^6^	9/12 (75)	<dl(9)	n.d.	128
6	MVA-N1-Ca	10^7^	4/12 (83)	<dl	n.d.	1024
7	rVVL-N1-Ca	10^6^	12/12 (100)	<dl	n.d.	1024
8	rVVL-N1-Ca	10^7^	12/12 (100)	<dl	n.d.	2048
9	Inact. H1N1(6)	3.75	11/12 (92)	640	538	256
10	MVA-wt(7)	10^7^	1/12 (8.3)	<dl	<dl	<2
11	VVL-wt(8)	10^7^	0/12 (0)	<dl	n.d.	<2
12	PBS	-	0/12 (0)	<dl	<dl	<2

(1) hemagglutinin-inhibition titer determined with chicken erythrocytes;

(2) geometric mean titer;

(3) microneutralization assay (cpe-based);

(4) neuraminidase-inhibition titer;

(5) two separate experiments with 6 animals per group were performed;

(6) inactivated whole virus vaccine, subcutaneous application route;

(7) wild-type MVA (NIH74 LVD clone 6);

(8) wild-type vaccinia Lister strain;

(9) the dectection limit (dl) is <10;

With the neuraminidase construct, MVA-N1-Ca, partial protection was achieved. Even in the unprotected mice the lung virus titers were rather low, indicating that some degree of protection was achieved also in these animals (**Fig. 2** **and** **Table 2**, groups 5–6). With the replicating vaccinia construct, rVVL-N1-Ca, full protection was seen at both doses (**Table 2**, groups 7–8). Neuraminidase-inhibiting antibodies were high after immunization with the viral constructs expressing NA, and absent in the HA constructs used as controls. As expected, the HI titers were below the detection limit. Mice immunized with the inactivated vaccine were almost fully protected with HI titers of 640 and NI titers of 256 (**Table 2**, group 9). Mice injected with the negative controls, empty MVA and VVL vectors and PBS, were not protected and sera were negative for HI and NI antibodies (groups 10–12). The number of protected mice in the groups immunized either with inactivated vaccine, or the live vaccines was significantly higher as compared to the controls (P<0.0001).

In addition, protection was also studied after **single dose** immunizations (dose 10^6^ TCID50 per animal) with the HA and NA constructs. As before, the challenge was carried out at day 42. In case of the HA viruses, 100% and 83% protection was achieved with the replicating vaccinia vectors and with the MVA-based vaccines, respectively. In case of the NA viruses, protection was less efficient, 33% and 67% protection was obtained with the replicating and the nonreplicating vectors, respectively.

### 3. Induction kinetics of antibody responses

Since induction of rapid immunity is crucial for pandemic vaccines, the kinetics of antibody induction against the major protective antigen, the hemagglutinin, was determined after single dose vaccinations. For this purpose, mice were immunized with increasing doses (range 10^6^ to 10^8^ pfu/animal) of the H1 containing vectors and with appropriate controls. Sera were taken each week over a period of 70 days, pooled by groups and analyzed using the HI- and µNT-test (**Table 3**). With *MVA-H1-Ca*, the first HI antibodies were detectable at day 7 (**Table 3** **, group 3**), at that time point the neutralizing antibodies were below the detection limit. Apparently independent of dose, both, neutralizing and HI antibodies were found after day 21. In the examined dose range, titers increased over time, however no clear dose dependence was seen. The titers induced by the replicating control, rVVL-H1-Ca, were clearly dose dependent. NT and HI titers were detectable starting at day 7 in the 10^8^ pfu dosage group (Table 3, group 6). Titers increased with time and remained high over the observation period. With the controls, MVA wt and PBS, the titers remained below the detection limit.

**Table 3:** Geometric mean neutralization (NT) and hemagglutination inhibitions (HI) titers of mouse sera after single dose immunizations with the live vectors.

Gr	Vaccine	Dose	Day 7 (1) [NT/HI]	Day 14 [NT/HI]	Day 21 [NT/HI]	Day 28 [NT/HI]	Day 35 [NT/HI]	Day 42 [NT/HI]	Day 70 [NT/HI]
1	MVA-**H1**-Ca	10^6^	<dl/<dl	<dl/14	80/40	57/67	320/80	160/113	453/226
2	MVA-**H1**-Ca	10^7^	<dl/<dl	<dl/10	40/40	28/57	160/80	160/95	320/160
3	MVA-**H1**-Ca	10^8^	<dl/10	20/20	80/80	113/113	226/160	160/160	640/320
4	rVV-L**-H1**-Ca	10^6^	<dl/<dl	<dl/<dl	57/28	40/57	80/113	57/80	453/160
5	rVV-L-**H1**-Ca	10^7^	<dl/40	40/57	20/80	160/113	160/226	640/226	1280/226
6	rVV-L-**H1**-Ca	10^8^	28/57	113/80	28/160	226/160	320/226	320/320	1810/320
7	MVA-wt(2)	10^6^	<dl/<dl	<dl/<dl	<dl/<dl	<dl/<dl	<dl/<dl	<dl/<dl	<dl/<dl
8	PBS	—	<dl/<dl	<dl/<dl	<dl/<dl	<dl/<dl	<dl/<dl	<dl/<dl	<dl/<dl

(1) day 0 titers were all below the detection limit (dl);

(2) wild-type MVA (NIH74 LVD clone 6); detection limits of the HI and the NT assay were <10.

### 4. Passive protection studies in severe combined immune-deficient (SCID) mice

Animal models should reflect the pathology induced in humans. In the case of the new H1N1 influenza, the model should protect from typical disease symptoms, such as malaise, pneumonia and even death. Since immune competent Balb/c mice and the slightly more susceptible CD1 mice recover from wt H1N1 challenge, SCID mice were used in the following experiments. For the passive protection experiments, first, antisera were generated. Immune competent mice were immunized twice with the MVA-H1-Ca and rVVL-H1-Ca vectors and sera were harvested at day 42. The HA-specific sera generated for transfer had HI titers of 640, while the titer of the control serum (MVA-wt vaccinated mice) was below the detection limit.

**Figure 3:**
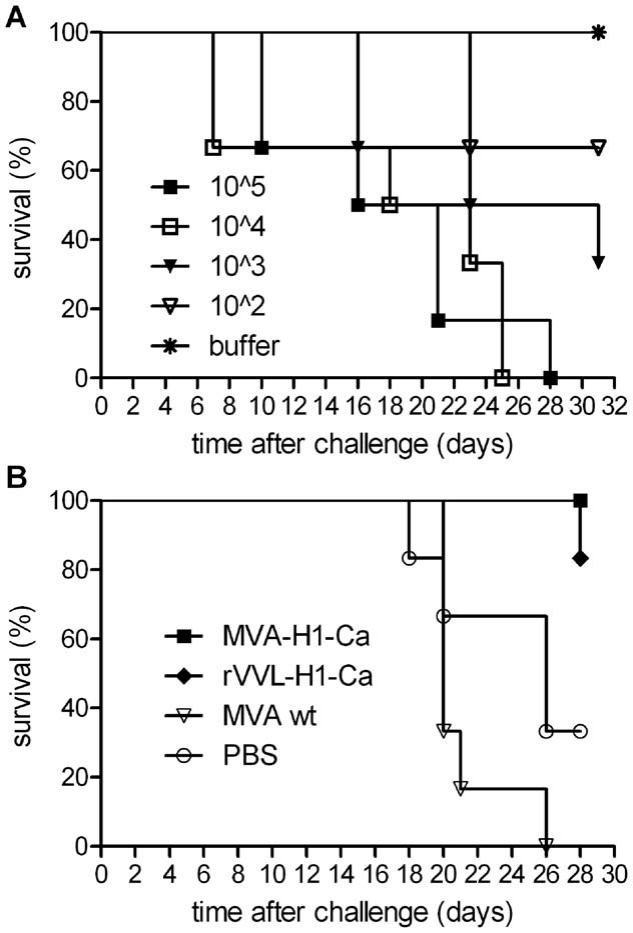
Survival of SCID mice after passive transfer of immune sera. A) Groups of three mice were infected intranasally with doses of H1N1 wild-type virus in the range of 10^2^ to 10^5^ TCID50 per animal and monitored over a 32 day period. The 10^4^ and 10^5^ doses were fully lethal. With the lower doses, mice survived the monitoring period. B) Passive transfer of mouse sera and challenge with a dose of 10^5^ TCID50 of H1N1 wild-type virus. All SCID mice receiving the sera of Balb/c mice vaccinated with MVA-H1-Ca were protected, while the MVA-wt controls all died.

The virulence of the new H1N1 strain in SCID mice was subsequently analyzed. After intranasal challenge with the CA/07 strain, the SCID mice died in a dose-dependent manner. Doses >10^4^ TCID50 per animal killed all mice within a four week period (**Fig. 3A**). In parallel, SCID mice were treated with the antisera. All mice treated with the MVA-H1-Ca induced sera were fully protected from death and disease symptoms while the survival rate of animals treated with the rVVL-H1-Ca-induced sera was 83%. The control animals, however, got sick and died in the third and fourth week after infection (**Fig. 3B**). In some of the animals protected by the MVA-H1-Ca induced sera, no virus was found in lungs at the end of the experiment at day 30. In others, despite lack of clinical symptoms, virus was present in the lungs (data not shown). Thus, passively transferred antibodies, induced by the MVA-H1-Ca live vaccine and the replicating controls, could fully protect mice from lethal challenge with the wild-type CA/07 virus confirming the important role of anti-H1 antibodies in protection from H1N1 influenza. Complete passive protection of SCID mice from lethal challenge by antisera generated in mice and guinea pigs with the inactivated H1N1 whole virus vaccine also used in this study was seen previously [16].

### 5. Hemagglutinin-specific and cross-reactive T cell responses in spleens

Subsequently, T cell responses induced by the different vaccines were characterized in mice immunized twice (days 0 and 21) with the MVA- and rVVL-H1-Ca and N1-Ca constructs. Splenocytes were prepared on day 28 and stimulated in-vitro with whole virus antigens or HA- or NA-specific peptide pools of overlapping 15-mers covering the entire HA or NA sequence of the CA/07 strain. The whole virus antigens used for stimulation included the monovalent vaccine bulks (MVBs) of inactivated influenza preparations of the H1N1 strains CA/07 (H1N1/CA), A/Brisbane/59/2007 (H1N1/BR), A/New Caledonia/20/99 (H1N1/NC) and the H5N1 strain A/Vietnam/1203/2004 (H5N1/VN). The frequencies of IFN-γ producing T cells were then determined by FACS-based intracellular cytokine assays. Differentiation between CD4 and CD8 T cells was achieved by staining with specific monoclonal antibodies (see methods).

**Figure 4:**
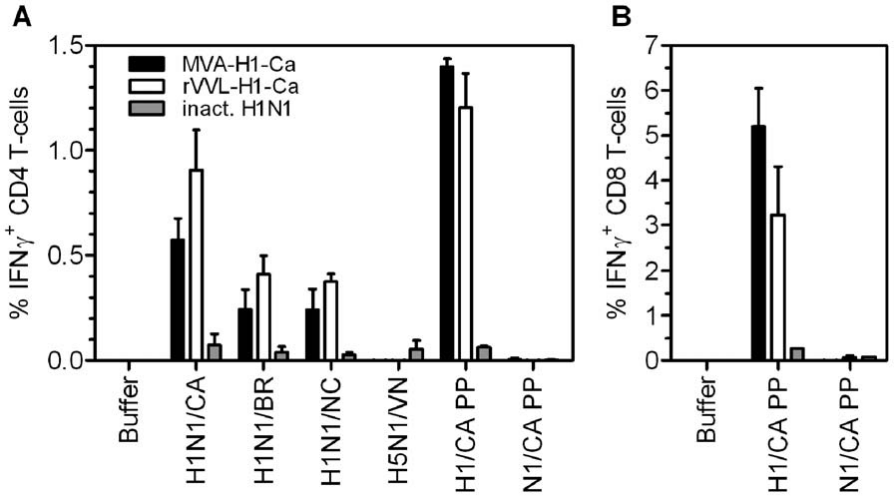
T cell induction by the hemagglutinin expressing live vaccines. **A**) Frequencies of influenza antigen specific IFN-γ+ CD4 T cells after immunizing two times with hemagglutinin constructs MVA-H1-Ca (black bars), rVVL-H1-Ca (white bars) or inactivated vaccine (grey bars) and stimulation with different antigens and peptides (shown on x-axis). Splenocytes were stimulated with buffer and with protein antigens (formalin-inactivated monovalent bulk material) of the influenza strains H1N1 California (H1N1/CA), H1N1 Brisbane (H1N1/BR), H1N1 New Caledonia (H1N1/NC), H5N1 Vietnam 1203 (H5N1/VN) and with peptide pools of overlapping 15-mer peptides of the swine flu hemagglutinin (H1/CA PP) or neuraminidase (N1/CA PP) antigens. **B**) Frequencies of influenza hemagglutinin-specific IFN-γ+ CD8 T-cells after two dosages of vaccine. Splenocytes were stimulated with the peptide pools indicated above. The data are mean values (+/− SEM) of two independent experiments.

The results obtained with hemagglutinin-containing constructs MVA-H1-Ca and rVVL-H1-Ca and with the inactivated vaccine are shown in **Fig. 4**. Specific induction of 0.5–0.8% of **CD4 T cells** was seen after homologous stimulation with H1N1/CA antigen of splenocytes of mice vaccinated with the MVA and VVL-based vaccines. This specific induction of CD4 T cells was significantly higher compared to the MVA wild-type group (P<0.05). Significant levels of cross-reactive T-cells recognizing seasonal H1N1/BR and H1N1/NC antigen were also detected, whereas no cross-reaction with pandemic H5N1/VN was induced (P>0.05). When using the H1 CA/07 peptide pool for stimulation of the splenocytes, 1.3–1.4% of the CD4 T-cells produced IFN-γ secretion in mice immunized with both the MVA and the VVL-based HA constructs. As expected, the levels of influenza-specific CD4 T-cells induced by the inactivated virus were several-fold lower (**Fig. 4A**).

Furthermore, CD8 T cells induced by the HA constructs were analyzed. The H1/CA peptide pool stimulated surprisingly high amounts of CTLs, around 3–5% of total CD8 T cells were specific for H1, whereas the N1-specific peptide pool used as a negative control, did not induce significant levels of CD8 T cells (P>0.05), **Fig. 4B**). MVA constructs induced slightly higher levels of CD8 T cells than the Lister constructs. The inactivated vaccine induced low but measurable amounts of CTLs. The splenocytes of mice vaccinated with the empty vectors did not react with the HA-specific antigens and peptides (not shown).

### 6. Neuraminidase-specific T cell responses in spleens

**Figure 5:**
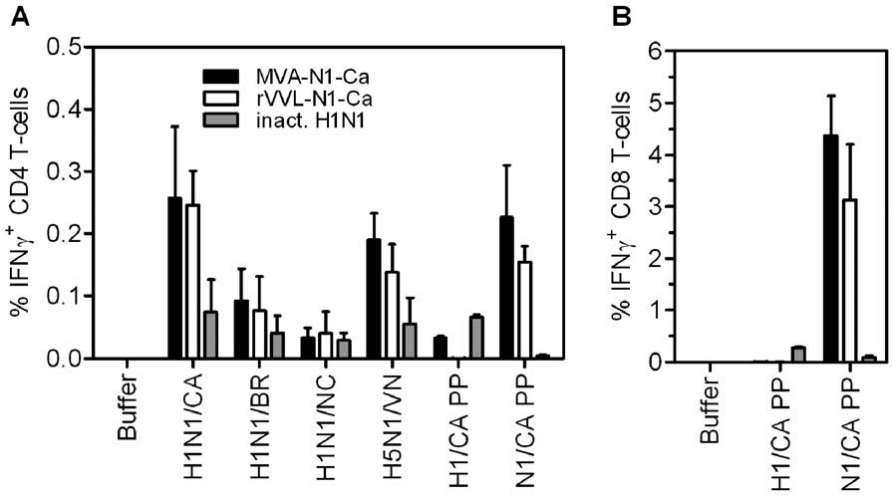
T cell induction by the neuraminidase expressing live vaccines. **A**) Frequencies of influenza antigen specific IFN-γ+ CD4 T cells after immunizing two times with MVA-N1-Ca (black bars), rVVL-N1-Ca (white bars) or inactivated vaccine (grey bars) and stimulation with buffer and with different antigens and peptides (shown on x-axis). Splenocytes were stimulated with protein antigens as described in the legend of Fig. 4. **B**) Frequencies of influenza neuraminidase-specific IFN-γ+ CD8 T-cells after two dosages of vaccine. Splenocytes were stimulated with the peptide pools indicated in the legend of Fig. 4. The data are mean values (+/− SEM) of two independent experiments.

To investigate neuraminidase-specific T cell responses, mice were immunized as described above with the neuraminidase-containing constructs MVA-N1-Ca and rVVL-N1-Ca and with the controls. The results obtained are shown in **Fig. 5**. Using the homologous stimulant, H1N1/CA antigen, induction of about 0.25% of CD4 T cells by the live vectors was achieved. With the Brisbane (H1N1/BR) and with the H5N1/VN antigens, good levels of cross reactivity were seen, while the New Caledonia H1N1 antigen (H1N1/NC) was a poor stimulant (**Fig. 5A**). A peptide pool of 15mers spanning the whole N1 sequence of CA/07 induced amounts of CD4 T cells comparable to the homologous proteinaceous antigen. Interestingly, a very strong CD8 T cell response against N1 was also observed in mice vaccinated with the N1-containing live vaccines (**Fig. 5B**). When using the N1 CA/07 peptide pool for stimulation of the cells, 3–4% of total CD8-T cells were NA-specific, whereas the H1 peptide pool, used as a negative control in this case, did not induce any IFN-γ response.

### 7. Hemagglutinin-specific T cell responses in the lung before and 3 days after intranasal challenge with H1N1 swine flu virus

To analyze whether the live vaccines, besides inducing high levels of influenza-specific CD4 and CD8 T-cells in the spleen shortly after booster immunization, also give rise to significant levels of HA-specific T-cells at the site of influenza infection, influenza-specific interferon *γ*-secreting T cells were also quantified in the lungs. Mice (5 per group) were immunized twice (at days 0 and 21) with the hemagglutinin constructs (MVA-H1-Ca and rVVL-H1-Ca), and with the MVA wt control (10^6^ pfu per animal). The challenge with H1N1 wt virus was carried out at day 42. Subsequently the pre- and post challenge lung T cells were analysed. For comparison reasons, T-cell responses were also measured at the same time points in the spleens, a site not directly involved in viral infection.

**Figure 6:**
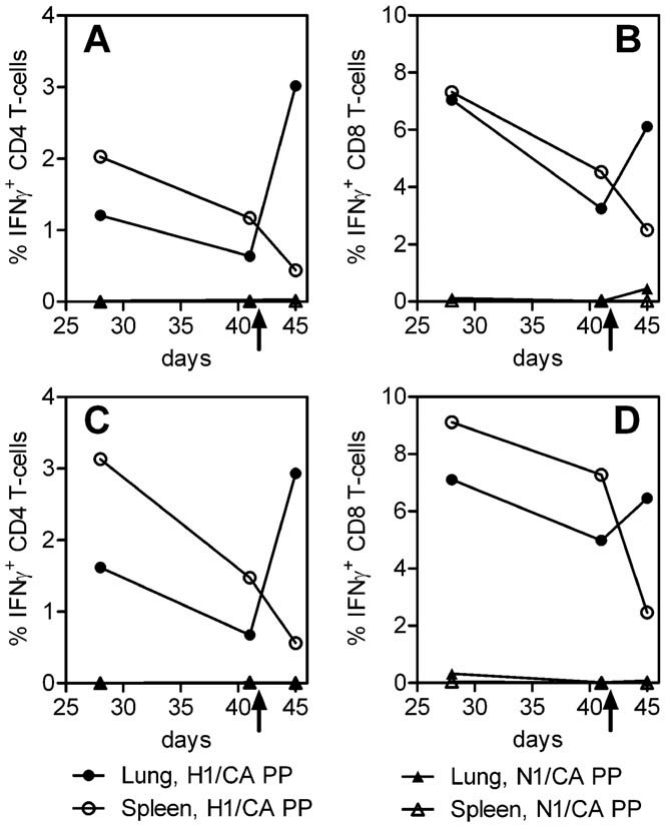
T cell induction by the hemagglutinin expressing live vaccines in the lungs. Frequencies of influenza antigen specific IFN-γ+ CD4 T-cells (**A, C**) and CD8 T-cells (**B, D**) after immunizing two times with hemagglutinin constructs MVA-H1-Ca (**A, B**) or rVVL-H1-Ca (**C, D**) and stimulation with hemagglutinin (H1/CA-PP) or neuraminidase (N1/CA-PP) peptide pools. Lung cells or spleen cells were isolated at days 28, 41 and 45. Animals were challenged with wild-type swine flu virus on day 42, as indicated by the arrow. The filled (open) circles indicate lung (spleen) cells stimulated with the hemagglutinin peptide pool H1/CA PP. The filled (open) triangles indicate lung (spleen) cells stimulated with the neuraminidase peptide pool used as negative controls. Representative results of two independent experiments are shown.

Seven days after the second immunization, and one day before and three days after the challenge (at days 28, 41 and 44), lungs and spleens were processed. Mice were given an anesthesia, lungs were flushed in-vivo with a heparin-containing medium to remove blood cells (that might have an impact on lung-specific T cell counts) and lungs and spleens were collected. Single cell suspensions were prepared from 5 pooled lungs or spleens of immunized mice. Pulmonary and spleen cells were stimulated with the HA- and the control peptide pools (H1/CA PP and N1/CA PP, respectively) and subjected to FACS analysis. High frequencies of influenza-specific CD4 and CD8 cells were induced by vaccination with the MVA-based HA vaccine, not only in the spleens but also in the lungs, although the frequencies were slightly lower in the lungs (**Fig. 6**). Seven days after the booster immunization, approximately 1% of the lung CD4 T cells (**Fig. 6A**) and 7% of the lung CD8 T-cells (**Fig. 6B**) were HA-specific. Two weeks later, levels of T-cells had consistently dropped by 40–50% in both lungs and spleens. Interestingly, however, three days after the challenge, a five-fold increase in IFN-γ expressing HA-specific CD4 T cells was found only in the lungs. In contrast, depletion of the spleens from influenza specific CD4 T-cells was observed at that time point, within three days after infection CD4 frequencies have dropped by 60% (**Fig. 6A**). A similar picture was seen for the CD8 T cells. Before challenge, about 3% of the CD8 pulmonary T cells were HA-specific, and a two-fold increase was detected after challenge with H1N1 wt virus at day 45, whereas the CD8 T-cell frequency in the spleens decreased by 50%. The NA peptide pool (N1/CA PP), used as a control in this experiment, induced only background levels of T cells, demonstrating that little or no T-cells induced by the infection with the swine virus were present in the lungs three days after challenge. Therefore, the observed increase of influenza-specific T-cells in the lungs upon challenge presumably reflects influx of the HA-specific effector T-cells from other sites into the lung.

Consistent results were obtained in mice immunized with rVV-H1-Ca live vaccine. Also in this group, a pronounced rise in influenza-specific CD4- (**Fig. 6C**) and CD8 T-cells (**Fig. 6D**) was observed after challenge with swine influenza virus. As expected, immunization with the MVA wt control did not induce any influenza-specific T-cells in the lungs before and after challenge. This clearly demonstrates that functionally competent effector T-cells were induced by the vaccine which were capable of homing into virus-infected tissue such as the lungs.

## Discussion

Nonreplicating poxviruses have been used recently as potential pandemic live vaccines against H5N1 influenza [23], [24], [25], [26], [27], [28]. In the current study, experimental vaccinia-based pandemic H1N1 influenza live vaccines expressing either the hemaglutinin or the neuraminidase of the novel swine H1N1 influenza strain were analysed. The hemagglutinin containing vaccines were almost fully protective in active immunizations, and protected animals had high pre-challenge HI and neutralizing antibody titers against the wild-type A/California/07/2009(H1N1) strain. The neuraminidase containing MVA viruses were partially protective and neuraminidase-inhibiting antibodies were detectable, consistent with earlier findings indicating that anti-NA antibodies do not neutralize influenza virus but interfere with virus release from the host cells resulting in reduction of titers [29]. After single dose vaccinations only the HA-containing live vaccines induced protection from virus in the lungs, presumably supported also by the strong T cell responses induced by these vaccines. Since both antigens contributed substantially to immunity, a bivalent vector might be the preferred choice as a pandemic vaccine.

Clear evidence exists that both CD4 and CD8 T cells contribute to viral clearance of influenza in the lungs of mice and thus contribute to protection [30], [31] and more recent work in chicken suggests, that pulmonary cellular immunity is very important in protecting naive natural hosts against lethal H5N1 influenza viruses [32]. T-cell responses to natural influenza infections are mainly directed against common epitopes on the nucleoprotein, PB2 and M1 but also, to a lesser degree, against the highly variable hemagglutinin and the neuraminidase [2] and thus, in contrast to the generally type-specific protection of antibodies against HA and NA, have the potential to provide broad, heterosubtypic protection. Indeed, vaccines would be preferable which, besides inducing a strong and stable humoral immune response, also induce a strong anti-influenza cellular response [33].

The MVA-based live vaccines induced surprisingly high amounts of CD8 T cells, with both the HA and the NA constructs giving rise to 4–5% of total antigen-specific CD8 T cells in splenocytes 7 days after the second immunization. Further, the levels of HA antigen-specific INF-γ producing CD4 T cells were also higher after immunization with the live vaccines. Considerable levels of CD4 T cells cross-reacting with related H1 strains were also found, while cross-reaction with the H5 subtype was not seen. CD4-T cells induced by the recombinant N1 vectors also cross-reacted with neuraminidase from seasonal H1N1 strains, and, interestingly, from the avian VN1203 H5N1 strain.

Furthermore, high levels of HA-specific CD4 and CD8 T-cells were detected in the lungs of mice immunized with the MVA-HA vaccine construct at the time of challenge with wild type H1N1/CA virus, with a further accumulation of effector T-cells observed in the lungs three days after the challenge. Accumulation of specific effector T-cells in the lungs of infected mice has been shown to depend on differential expression of homing receptors such as CD44 or CD62L [34], which target the cells from the lymphoid tissue to non-lymphoid organs. However, upon pulmonary infection with influenza virus, IFN-γ secreting CD8 T-cells can be detected only after 5 days in the lungs [35] whereas, after immunization with MVA-H1, they are present already in high frequencies before infection, and, upon infection, enter the lungs much more rapidly by relocation from other sites such as the spleen. These influenza-specific effector T-cells present at the site of infection have the potential to contribute to protection in various ways, for instance, by inhibition of viral replication, by secretion of cytokines, by direct killing of virus-infected host cells, or by support of influenza specific B-cells. Interestingly, in Balb/c mice, the primary pulmonary CD8 T cell response against influenza A/Japan/305/57(H2N2) was mainly directed against three epitopes in the HA and one in the NP [35], suggesting that CD8 T cell responses are strongly dependent on the infecting virus strain and the host genetic background. Moreover, lung-resident proliferation contributes significantly to the magnitude of the Ag-specific CD8 T cell response following influenza virus infection [36].

A further advantage of using MVA as pandemic influenza vaccine is its genetic stability including that of the expressed foreign genes. Passage of influenza primary isolates in eggs usually results in adaptive mutations in the HA gene. When non egg-adapted human influenza virus, i.e., either the natural virus present in a clinical specimen or an isolate propagated exclusively in tissue culture cells, is first passaged in the allantoic cavity of embryonated hens' eggs, variants which have amino acid substitutions around the receptor binding site are selected [37]. Therefore, egg adaption can result in altered antigenicity which can result in reduced protection [2]. To preserve the original genotype of viruses used as reference strains, a complex procedure is recommended, involving cloning in chicken eggs of the candidate virus at a very early passage, selection and analysis with appropriate antibodies and selection of the isolate whose hemagglutinin molecule most closely resembles the clinical isolate [38]. All these complications are overcome if a stable DNA virus, such as MVA, is used as a vector for the influenza genes. This vector is independent of influenza virus-specific selection mechanisms and thus, the originally inserted sequences do not change resulting in preservation of the originally inserted HA or NA genes.

MVA-based pandemic vaccines may also have advantages as compared to cold-adapted live influenza vaccines. In a recent evaluation of live attenuated cold-adapted H5N1 influenza virus vaccines in healthy adults, HI and neutralizing antibody responses were found to be minimal [8]. Several reasons were identified for this failure: the attenuating mutations specified by the A/Ann Arbor/6/60 (H2N2) cold-adapted virus loci had the greatest influence, followed by the deletion of the H5 HA multi-basic cleavage site (MBS) and the constellation effects of the internal backbone genes acting in concert with the H5N1 glycoproteins [9]. In poxviral vectors, these influenza virus-specific effects do not play a role. Injection of defined amounts of a stable vector results in reliable delivery, moreover the use of strong vaccinia promoters and optimized foreign genes result in high-level expression and good induction of B and T cell responses. In addition, MVA tolerates pre-existing anti-vaccinia immunity and can be used as an immunizing agent under conditions of pre-existing immunity to the vector and thereby may allow repeated use [39].

Furthermore, full protection of SCID mice was achieved after passive transfer of sera from MVA-H1-Ca immunized mice while the control mice, challenged with a dose of 1×10∧5 TCID50 of wt CA/07 virus died within three weeks from pneumonia with titers of 5–6 log10 TCID50 being found in the lungs. Interestingly, despite lack of clinical symptoms in the protected SCID mice, virus was found at the end of the 4 week monitoring period in the majority of lungs. This indicates that a single prophylactic dose of antiserum given prior to challenge was not sufficient for virus clearance. However, as shown previously by others [40], infection of SCID mice with A/PR8/34(H1N1) influenza virus followed by passive transfer of two doses of an anti-hemagglutinin antibody cocktail at days 2 and 6 had lead to clearance and full recovery from infection. Thus, protection including clearance of virus, may be achieved by a higher dose and by repeated dosing of the antiserum. Furthermore, in the case of H1N1 CA/07 virus, full protection of SCID mice was obtained after passive transfer of sera generated with the inactivated whole virus vaccine in mice and guinea pigs [16].

In conclusion, the mouse data presented here suggest that modified vaccinia Ankara-based recombinant viruses, besides demonstrated safety, have some further advantages as pandemic influenza vaccines, including excellent induction of both antibody and T cell responses, genetic stability of the hemagglutinin due to independence from influenza-specific genetic alterations and efficacy after single dose vaccination. Our study shows that MVA is a good alternative as pandemic influenza vaccine against the novel H1N1 subtype, in accordance with previous studies demonstrating that corresponding MVA recombinants also protect against avian H5N1 strains [23], [24]. The H1N1 pandemic strain currently causes mild disease presumably due to partial immunity mainly in the adult population. If - similar to the 1918 pandemic - more virulent mutants would occur in subsequent waves of infection, more broadly protecting vaccines would be desirable.
